# The health and economic burden of dust pollution in the textile industry of Faisalabad, Pakistan

**DOI:** 10.1186/s42506-024-00150-2

**Published:** 2024-01-29

**Authors:** Muhammad Khan, Kashif Muhmood, Hafiz Zahid Mahmood, Imran Hameed Khaliq, Shakila Zaman

**Affiliations:** 1 Department of Economics, COMSATS University Islamabad, Lahore Campus, Lahore, Pakistan; 2 University of Health Sciences, Lahore, Pakistan

**Keywords:** Textile industry, Cotton dust exposure, Respiratory disorders, Medical records, Sickness absence, Q52, Q53, I

## Abstract

**Background:**

Exposure to dust in textile mills adversely affects workers’ health. We collected epidemiological data on textile workers suffering from respiratory diseases and assessed work absence associated with illnesses in Faisalabad, Pakistan.

**Methods:**

We recruited 206 workers using multistage sampling from 11 spinning mills in Faisalabad, Pakistan. The data were collected using 2-week health diaries and face-to-face interviews. The data pertains to socio-demographics, occupational exposures, the state of the workers’ health, and other attributes. A theoretical framework of the health production function was used to estimate the relationship between cotton dust exposure and respiratory illnesses. We also estimated functional limitations (e.g., work absence) associated with dust exposure. STATA 12 was used to calculate descriptive statistics, an ordered probit for byssinosis, a probit model for chronic cough, and three complementary log-log models for blood phlegm, bronchitis, and asthma to measure dose–response functions. A Tobit model was used to measure the sickness absence function.

**Results:**

We found that cotton dust exposure causes a significant health burden to workers, such as cough (35%), bronchitis (17%), and different grades of byssinosis symptoms (22%). The regression analysis showed that smoking cigarettes and working in dusty sections were the main determinants of respiratory diseases. Dusty work sections also cause illness-related work absences. However, the probability of work absence decreases with the increased use of face masks.

**Conclusion:**

The study’s findings imply the significance of promoting occupational safety and health culture through training and awareness among workers or implementing the use of safety gadgets. Promulgating appropriate dust standards in textile mills is also a need of the hour.

**Supplementary Information:**

The online version contains supplementary material available at 10.1186/s42506-024-00150-2.

## Introduction

According to the Joint Statement of the World Health Organization (WHO) and the International Labor Organization (ILO), approximately 2 million workers died from occupational hazards in 2016 at the global level [1]. Work-related diseases majorly contribute to workers’ deaths, followed by occupational injuries. Occupational injuries and illnesses come with a massive economic burden and constitute massive social costs. According to an ILO report (2003), the economic costs of work-associated sick leaves, compensation for work injuries, production interruptions, and medical expenses account for 4% of the annual world gross domestic product (GDP), amounting to USD 2.25 trillion [2].

The textile sector is among the most labor-intensive industries, employing approximately 60 million workers globally [3]. Generally, the typical textile industry comprises different segments: spinning, weaving, processing, bleaching, dyeing and finishing, and stitching. Workers associated with the textile industry, especially those working in highly exposed sections, e.g., in the spinning segment, are at high risk of inhaling a large amount of cotton dust, likely affecting their lungs’ function [4]. The burden of occupational diseases is directly associated with such exposure. At the same time, chronic exposures can lead workers to suffer from byssinosis or fatal diseases [5, 6]. The typical symptoms of byssinosis include but are not limited to chest tightness, breathlessness, cough, tuberculosis, asthma, and phlegm. However, the type and severity of the problems usually depend on the intensity and the exposure period [7–10].

A dose–response relationship is well documented between respiratory symptoms and working conditions in the textile industry [5, 10, 11]. Advances in dust control measures have assisted developed countries in lessening the prevalence of respiratory symptoms and byssinosis among textile industries. For example, a UK-based study reported that only 3% of workers in the spinning segment had byssinosis., This number was relatively minor (0.3%) among workers in the weaving segment [12]. In contrast, developing countries have an alarming situation where the disease is common in African and Asian countries, e.g., Ethiopia (46%) [11], India (12%) [13], Pakistan (16%) [6], and Benin (21.1%) [14].

The textile industry of Pakistan has a large manufacturing sector that employs a significant proportion of the workforce by contributing approximately 8% to the country’s GDP and 58.98% to export earnings [15]. This sector also concentrates on the spinning segment, which enables the sector to export a significant proportion of good-quality yarn. Regardless of the pivotal role of the textile sector in the economic growth of the country, it is considered the most polluting domestic industry [16]. Unfortunately, the country lacks sector-wise disease-specific updated data, but according to an old estimate, over 0.8 million textile workers are routinely exposed to cotton dust [17].

In Pakistan, evidence suggests a significant relationship between the respiratory symptoms of textile workers and their exposure to cotton dust in the workplace. Many studies have also reported moderate to high (ranging from 8 to 35%) levels of byssinosis among textile workers with a high percentage of chronic respiratory morbidities, including bronchial asthma, chronic bronchitis, tuberculosis, and other obstructive pulmonary symptoms [5, 6, 8, 10, 18–21]. Although a relationship exists between cotton dust and its effects on workers' health, there is a shortage of information on the economic valuation of dust pollution that directly affects workers' health, productivity, and quality of life. Such information is also vital for policymakers, enabling them to implement dust standards for matters related to cotton dust pollution and propose the allocation of resources for the welfare of workers. The textile industry of Pakistan is a model country to conduct this research, whose findings may be generalized beyond the geographical boundaries since it represents nearly 15 million global laborers in the textile sectors [8]. The objectives of this study were to record detailed epidemiological data on textile workers suffering from respiratory diseases and to assess the economic burden of health problems arising from dust pollution among textile workers.

## Methods

### Study setting

This study was conducted in Pakistan’s third-largest industrial zone, Faisalabad District. Of the 612 large-scale industries in the district, nearly 40% are textile and garment industries [22]. The heavy concentration of industries depicts high participation in the labor force [23]. Unfortunately, air quality in the city is not yet monitored, and industrial centers have no wastewater treatment facilities [22].

### Participant recruitment and consent to participate

In total, 210 workers from 11 spinning mills were randomly selected who met the inclusion and exclusion criteria. Workers were eligible if they were 18 or older, were employed in the textile industry over the past 2 years, and could comprehend and communicate in local languages [24]. However, textile workers who did not provide written informed consent were barred from participating in the study. Additionally, translated leaflets in the local language (i.e., Urdu) were provided and verbally guided before the data collection to the workers, informing them of the purpose of the research, ethical approval, and confidentiality of the participants’ details.

### Development of study instruments

We used two instruments to collect data: health diaries and workers’ respiratory health surveys. Health record-keeping diaries are used to collect data due to their higher accuracy, fewer recall problems, better sequencing capabilities, and the ability to capture low-profile events. A validated health diary was adopted from Usha Gupta (2008) and Naveen Adhikari (2012) [25, 26]. We used a modified version of the health diary to collect detailed data on the state of workers’ health along with potentially harmful consequences such as lost workdays and medical expenditures associated with ill health. Each worker was given a health diary to complete for 15 consecutive nights. After the prescribed time, 206 workers (out of 210 workers) returned completed diaries. The workers who returned the health diaries were qualified for face-to-face interviews.

Face-to-face interviews were based on the Workers’ Respiratory Health Questionnaire. The validated Respiratory Health Questionnaire was taken (on request) from Professor David C. Christiani from Harvard T. H. CHAN School of Public Health. The same questionnaire was used in the study of Chinese textile mill workers [27]. The survey comprised workers’ information regarding socio-demographics, occupational exposures, chronic diseases, etc. The questionnaire validation statistics show that the scales are reliable. The Cronbach’s alpha test score is 0.7762, which is a generally acceptable score.

The data were collected in August and September 2013. The study instruments (health diary and survey questionnaire) were pretested on twenty workers of a textile spinning mill. The questionnaire required a few modifications before the final data collection based on the pretest experience.

### Sample size and sampling technique

Data on spinning mills in the district of Faisalabad were retrieved from the All-Pakistan Textile Mills Association (APTMA). The total number of spinning mills in the district was 46. Faisalabad District is divided into six tehsils (subdistricts), namely, Tehsil Saddar, Tehsil Jaranwala, Tehsil Chak Jhumra, Tehsil Khurrianwala, Tehsil Tandianwali, and Tehsil Samundary. To ensure representation of the population from every Tehsil, we short-listed spinning mills by Tehsil.

Stage 1: Based on the distribution of mills by tehsils, we purposively decided to select two mills from three Tehsils (Jaranwala, Chak Jhumra, Tandianwali), one mill from Tehsil Samundary and Tehsil Saddar (because of the low concentration of textile mills there), and three mills from Tehsil Khurrianwala (because of the high concentration of textile mills there).

Stage 2: We then started the randomization process to select the desired data. Therefore, using the “=RAND ()” command in Excel Spreadsheet, 11 spinning mills were randomly selected across tehsils.

Stage 3: In this stage, 15 to 25 workers representing each section of the spinning mill were purposively selected from sample mills based on the workforce size. Later, an informed worker (called a ‘monitor’) from each textile mill was chosen to help the research team contact workers to participate in the study and remind workers every day to complete a health diary. The monitors also maintained contact between the data enumerators and the sample workers.

### The theoretical model

This study used a health production function (HPF) as a theoretical framework. The household health production function is a work of Grossman, which was expanded by Harrington and Portney. The health production function is complex and dynamic and incorporates an individual willingness to pay (“investment in human capital”) to increase an individual's utility in the form of reduced illness over several periods. Individuals maximize their utility by selecting an optimal combination of demand functions for averting and mitigating activities [28]. Therefore, estimating a health production function can be complex due to the multifaceted and interrelated nature of health determinants. Additionally, data availability and quality can influence the accuracy of the estimates. As a result, many studies in recent times, such as Khan and Lohano (2018) [28], used a simplified version of the health production function described in Freeman et al. 2014 [29] (see pages 214 and 215).

Under this framework, a person’s health status depends on exogenous variables (exposure to pollution), choice variables (e.g., averting actions), and other characteristics related to physical and socioeconomic conditions. We also used the simple model described by Freeman et al. (2014; 214) [29]. Suppose health is represented by the number of sick days at any time (15 days in the current study) and is denoted by S.

1 $$S=S\left(D,G\right)$$

where ***S*** is sickness days (an indicator of health status); ***D*** represents the dose of pollution; and ***G*** represents demographic and personal characteristics. The level of pollution exposure or dose D and demographic characteristics *G* determine the health *S*. The dose *D* is a scaler variable that depends on the concentration of pollution or contaminant, *C* (if the contaminant is air pollution, C could be interpreted as the number of days during which some measure of air pollution exceeds the stated standard), and averting activities *A*, to reduce exposure such as the use of face masks. Hence, *D* depends on pollution concentration *C* and Averting actions *A* (e.g., use of mask), as shown in Eq. 2:

2 $$D=D\left(C,A\right)$$

Substituting Eq. (2) for Eq. (1) gives Eq. 3:

3 $$S=S\left(C,A,G\right)$$

with $$\frac{\partial S}{\partial C}>0,$$ $$\frac{\partial S}{\partial A}<0,$$ $$\frac{\partial S}{\partial B}<0$$

In Eq. (3), the “dose–response function” shows the relationship between cotton dust exposure and health status. The dose–response function requires collecting physical data on factory conditions (i.e., suspended cotton dust) and medical examination of workers’ health status. These data can then associate an illness with a specific agent.

The consideration of collaborating physical evidence is affected by resource constraints and lack of cooperation by most of the mill administrations in the sample mills. The literature on byssinosis can reasonably solve this problem. The literature has shown that a dose–response relationship has repeatedly been established between byssinosis in cotton textile workers and levels of dust in cotton mills. Most of the studies building upon the literature on the dose–response relationship focused only on the prevalence of disease in textile mills rather than using scientific instruments to collect dust data [6, 30].

It must be noted that over the last six decades, studies have been using self-reported data from textile workers about symptoms of byssinosis using standard questionnaires of Schilling’s grading methodology for diagnosing the disease [31]. Self-reported data are often followed by workers' medical examinations (spirometry or pulmonary function tests). Spirometry provides the additional advantage of diagnosing impaired lung function among those who do not have apparent symptoms, and it may not be possible to capture such impairment by using questionnaires alone [32–34].

A study by Jamali and Nafees compared the results of spirometry and byssinosis questionnaires in identifying byssinosis and respiratory diseases [34]. The results illustrated that self-reported respiratory symptoms identified by the questionnaire could be good predictors of impaired lung function, and the questionnaire could be used as a validated tool to estimate the burden of respiratory symptoms among the working population. Similar findings have been reported in previous studies [32, 33]. Therefore, both the Pulmonary Function Test (PFT) and byssinosis questionnaires are acceptable diagnostic criteria for byssinosis. This is the reason why a few studies used the PFT for diagnosing byssinosis, whereas the majority of the studies only applied Schilling’s grading methodology [6, 14, 18, 19].

Following the literature, the current study defines factory conditions by characterizing dust by work section. The literature shows the association of the disease with the work area. Many studies noted that byssinosis is significantly higher in earlier stages of the textile process, such as bale opening, blow room, and card room, because of the high concentration of dust in these areas [6, 11, 13, 14, 18, 19, 26]. In addition, we collected self-reported data on dust level, e.g., less dusty than normal to more dusty than normal.

Another potential problem in the study is the lack of standard diagnostic measures for byssinosis (e.g., relying on self-reported illness, which may correlate with workers' perception rather than physical exam). Again, literature has somewhat addressed this problem. In recent times, few studies have attempted to verify the results across different diagnostic criteria for byssinosis [18, 19]. These studies collected self-reported data from textile workers about symptoms of byssinosis using standard questionnaires of Schilling’s grading methodology for diagnosing the disease. Medical examinations of the workers then followed the self-reported data. The variation between results across techniques was negligible. Therefore, both PFT and standard byssinosis questionnaires are acceptable diagnostic criteria for byssinosis. In fact, most studies applied Schilling's grading methodology in the literature compared to fewer studies that used PFT for diagnosing byssinosis.

This study collects self-reported data (using a standard questionnaire) from the target respondents concerning the prevalence of byssinosis, chronic cough, phlegm, and blood with phlegm. For bronchitis, asthma, and tuberculosis, we asked workers to report whether the healthcare providers diagnosed these conditions.

### Measurement of byssinosis

A standard tool for measuring byssinosis was adopted for this study, i.e., a byssinosis questionnaire using Schilling’s classification (grading) criteria, which various studies have previously used to diagnose byssinosis [18, 19, 30]. Thus, byssinosis was graded as grade 0: no symptoms of breathlessness or chest tightness on the opening day of work after the weekly break; grade ½: occasional breathlessness or chest tightness on the opening day of work after the weekly break; grade 1: breathlessness or chest tightness only on the opening day of work after the weekly break; grade 2: breathlessness or chest tightness on the opening day of work after the weekly break as well as on other weekdays; grade 3: evidence of permanent impairment in capacity from reduced ventilator defect along with grade 2 symptoms [31].

### The estimation methods

Various dose–response functions were estimated using the appropriate regression models. Equation (3) can be rewritten in the following econometric form:

4 $$S=S\left(C,A,G\right)+\varepsilon$$

The dependent variable *S* indicates the following respiratory diseases: byssinosis, asthma, blood phlegm, chronic cough, and bronchitis. For byssinosis, the dependent variable is defined as the categorical variable, which includes values from 0, 1, 2, 3: i.e., 0 if there is grade 0 byssinosis (no byssinosis), 1 if grade ½ byssinosis, 2 if grade 1 byssinosis and 3 if grade 2 and 3 byssinosis. Since values of byssinosis are in ordered form, an ordered probit model is used for the analysis. The dependent variable is binary for the rest of the diseases. Therefore, probit models are appropriate for use in other diseases. However, blood phlegm, asthma, and bronchitis data were not normally distributed. Therefore, the probit model is used for the chronic cough variable only. For other diseases, complementary log-log regression is used. Complementary log-log regression is an alternative to logit or probit when data are not normally distributed. The definitions of all the independent variables can be found in Supplementary Table.

### Statistical analysis

The statistical package STATA 12 (Stata Corp LLC TX, USA) was used to estimate the dose–response function. Before model estimation, descriptive statistics of key variables were provided. We estimated an ordered probit model for byssinosis, a probit model for chronic cough, and three complementary log-log regression models for blood with phlegm, bronchitis, and asthma.

## Results

Table 1 shows that most workers were males (86%) compared to their female counterparts (14%). The average age of the workers was 28 ± 6.94 years. The average wage of workers amounted to PKR 13,392 ± 5734. Furthermore, the average wage of female workers was 30% lower (PKR 9,857) than that of male workers (PKR 13,949). Only 14% of workers reported using masks at the workplace. In response to a question, ‘Is the factory providing them with face masks?’, all the workers reported that they are provided face masks by the textile mills at no cost.

**Table 1:** Socio-demographic, diagnoses, and work-related characteristics of workers in Spinning mills, Faisalabad District, Pakistan, 2013

Variables	*N* (%)	Mean±SD
Dose–response variables (D)		
Byssinosis		
No byssinosis	160(77.67)	–
Occasional chest tightness on Monday	3(1.46)	–
Chest tightness on Monday only	16(7.77)	–
Chest tightness on other days as well as on Monday	27(13.11)	–
Chronic cough	74(35.92)	–
Blood phlegm	19(9.22)	–
Asthma	11(5.34)	–
Bronchitis	35(16.99)	–
Health production variable (S)		
Work hours lost		
Number of work hours missed in 15 days	–	2.37(6.66)
Averting activities variables (A)		
Use of mask	–	0.14(0.32)
Personal characteristics (G)		
Gender		
Male	178(86.41)	–
Female	28(13.59)	–
Age (years)	–	28.34(6.94)
Smoking cigarettes per day	–	3.49(6.85)
Marital status		
Married	127(61.65)	–
Unmarried	79(38.35)	–
Education (years)	–	7.85(3.20)
Income and job-related variables (I)		
The income per month (PKR)	–	20320(8653)
Wage per month (PKR)	–	13392(5734)
Permanent employee	62(30.10)	–
Casual employee	110(52.40)	–
Daily wage workers	34(16.50)	–
Factory or environmental characteristics (C)		
Working section		
Ring frame	26(12.62)	–
Bale Opening	47(22.82)	–
Blow room	26(12.62)	–
Card room	34(16.50)	–
Samplex	36(17.48)	–
Autocone	37(17.96)	–
Average temperature (C°)	–	32.8(0.60)

Table 1 further shows that 36% of workers complained of chronic cough, 9% blood with phlegm 4.4% asthma, and 17% at least one episode of bronchitis. Overall, 22.3% of workers reported that they experienced byssinosis. Regarding the classification of byssinosis by grade, it was found that 7.8% reported grade ½, 13.1% reported grade 1, and 1.4% reported grade 2 or 3.

### Results of the dose–response function

The dose–response function estimated the relationship between exposure to cotton dust and the development of respiratory diseases among textile workers. The ordered probit model and the marginal effects were calculated, Pseudo *R*^2^ = 0.106 (Table 2). The results showed that work sections and temperature were significant among the environmental (pollution) and factory characteristic variables. The results showed that workers working in dusty or above-average temperature sections were more likely to develop byssinosis than workers working in less dusty and normal temperature sections. The marginal effect of the work section dummy indicated that if the worker leaves from the ring (or base) section (a relatively less dusty section) and joins the opening section, the probability of developing different grades of byssinosis increases by 14% for grade ½, 7% for grade 1 and 9% for grade 2 or 3.

**Table 2:** Marginal effects of the status of illness (ordered probit) (*N* = 206)

	Dependent variable: byssinosis				
Independent variables	Coefficient	Marginal effects			
		No byssinosis	Grade 1/2	Grade 1	Grades 2 and 3
Environmental characteristics (C)					
Opening	0.951**	− 0.304**	0.141**	0.070**	0.093**
Blow room	1.400**	− 0.487**	0.167**	0.111**	0.208**
Card room	1.420**	− 0.488**	0.174**	0.112**	0.202**
Simplex	1.012*	− 0.336*	0.146*	0.078*	0.111*
Autocone	0.962 *	− 0.317*	0.141*	0.073*	0.101*
Dust level in section	0.342	− 0.092	0.053	0.020	0.019
Temperature in section	0.459**	− 0.124**	0.029**	0.026**	0.026**
Averting activities (A)					
Use of mask	0.266	− 0.078	0.042	0.017	0.018
Personal characteristics (G)					
Gender (male)	− 0.136	0.038	− 0.021	− 0.008	− 0.008
Age	0.023	− 0.006	0.003	0.001	0.001
Smoking	0.034**	− 0.009**	0.005**	0.001**	0.001**
Marital status (married)	− 0.024	0.006	− 0.003	− 0.001	− 0.001
Pseudo R squared	0.1061				

The significance levels at 1%, and 10% are denoted by **, and *, respectively

Similarly, if the worker leaves the ring section and joins the blow room section, the probability of developing different grades of byssinosis increases by 16% for grade 1/2, 11% for grade 1, and 20% for grade 2 or 3. Similarly, if an employee moves from the ring section to the card room department, the chances of developing different grades of byssinosis increase by 17% for grade ½, 11% for grade 1, and 20% for grade 2 or 3. The marginal effect of temperature indicates that an increase in temperature by 1 centigrade in the workplace increases the probability of developing byssinosis of grade 1/2 by 0.029%, byssinosis of grade 1 by 0.026%, and byssinosis of grade 2 or 3 by 0.026%.

Among personal characteristics, smoking was a highly significant (at 1%) determinant of byssinosis. The marginal effects of the variable showed that smoking one additional cigarette per day increases the probability of developing byssinosis of grade 1/2 by 0.5%, byssinosis of grade 1 by 0.2%, and byssinosis of grade 2 or 3 by 0.2%.

Table 3 shows the probit regression results for chronic cough and complementary log-log regression results for other respiratory diseases, such as asthma, blood phlegm, and bronchitis. The dust level within the environmental and factory characteristics variables was positively and significantly associated with blood phlegm and bronchitis. Again, the results show that respiratory diseases, chronic cough, and bronchitis are more prevalent in dusty work sections, e.g., blow rooms, card rooms, and opening sections, than in less dusty sections. The temperature coefficient was positive, indicating that the incidence of cough increased as the temperature increased. The results of environmental and factory characteristic variables show that there is a clear association between dust exposure and respiratory illnesses in textile factories.

**Table 3:** Marginal effects of the status of illness (*N* = 206)

	Dependent variables			
Independent variables	Chronic cough	Blood phlegm	Asthma	Bronchitis
Environmental and factory characteristics (C)				
Opening section	0.10 (0.135)	06.07 (16.70)	− 0.853(1.037)	1.191 (1.041)^c^
Blow room section	0.343 (0.137)^a^	0.103 (2.36)	0.6000(0.852)	1.587 (1.087)
Card room section	0.241 (0.138)^c^	0.968 (3.27)	0.442(0.790)	1.771 (1.081)^c^
Simplex section	0.110 (0.137)	0.101 (10.67)	0 omitted	1.444 (1.085)
Autocone section	− 0.061 (0.134)	0.936 (17.5)	− 2.156(1.426)	1.113 (1.126)
Dust level in section	0.029 (0.117)	0.060 (3.12)^a^	1.002 (1.061)	1.214 (1.082)^c^
Temperature in section	0.163 (0.058)^a^	0.013 (0.716)	1.111(1.093)	1.075 (1.052)
Averting activities (A)				
Use of mask	0.048 (0.118)	0.074 (0.405)	0.970(0.563)	− 0.120 (0.284)
Personal characteristics (G)				
Gender (male)	− 0.127 (0.122)	− 0.913 (0.720)	− 1.984 (0.985)^b^	− 0.002 (0.001)
Age	.003 (0.005)	− 0.075 (0.454)	− 0.421(0.565)	− 0.042 (0.024)
Marital status(married)	− 0.079 (0.494)	− 0.005 (0.302)	− 0.267(.643)	− 0.003 (0.004)
Smoking	0.002 (0.005)	0.005 (0.002)^a^	0.004(0.003)^c^	0.004 (0.001)^a^
Constant	− 15.390^a^	− 18.03	− 1.673(1.902)	− 2.699 (1.362)^b^
Pseudo R squared/LR Chi^2^	0.080	0.082	0.154	0.151

The significance levels at 1%, 5%, and 10% are denoted by ^a^, ^b^, and ^c^, respectively. The values in parentheses are standard errors

Within personal factors, the results showed that cigarette smoking was positively associated with asthma, blood phlegm, and bronchitis, and the results were significant at the 1% level for blood phlegm and bronchitis. The results also indicated the role of sex in the development of asthma. For example, the estimates showed that the probability of developing asthma is significantly higher among female workers than male workers.

### Results of the sickness absence function

Within the environmental and factory aspects, the coefficients of all the variables were positive with sickness absences (Table 4). Only two variables, temperature, and blow room section, were not significant. The marginal effects of dustiness showed that an increase in the level of dustiness of the mill by one stage leads to the loss of 5 h of work. Specifically, the result of marginal effects indicated that when a worker moves from the ring (base) section to the opening section, the chances of work absence increase by 3 work hours. Similarly, if a worker moves from the ring section to the card room section, the chances of work absence increase by 2.8 work hours. As in, if a worker moves from the ring section to the samplex section or Auto cone section, the chances of work absence increase by 2.65 and 3.4 work hours, respectively.

The marginal effects of the use of the mask (averting action) variable showed a negative association with work hours lost, and the result was significant at the 10% level. Specifically, the marginal effect showed the probability of work hours lost by 2.4 h if workers wore a mask during work compared to workers who did not use the mask.

**Table 4:** Marginal effects of sickness absence function (Tobit Model) (*N* = 206)

Independent variables	Dependent variable: work hours lost
	Marginal effects
Environmental or factory characteristics (C)	
Opening	3.031^b^ (7.794)
Blow room	1.999 (8.441)
Cardroom	2.824^c^ (7.956)
Samplex	2.652^c^ (7.879)
Auto cone	3.392^b^ (8.026)
Dust level	4.940^a^ (6.086)
Temperature	.0964 (2.694)
Averting activities (A)	
Use of mask	− 2.430088^c^ (6.689)
Mitigating activities (B)	
Visit doctor	1.673862 (5.749)
Personal characteristics (G)	
Gender (male)	− 1.248 (5.297)
Age	0.030 (0.2768)
Marital status (married)	0.026 (3.557)
Smoking	0.012 (0.268)
Pseudo R squared	0.0613

The significance levels at 1%, 5%, and 10% are denoted by ^a^, ^b^, and ^c^, respectively. The values in parentheses are standard errors

## Discussion

The results of this study provided evidence of respiratory impairments such as byssinosis, chronic cough, bronchitis, asthma, and blood with phlegm among textile workers. The analysis suggests that the prevalence of these illnesses is associated with dust concentration in the work environment. Cigarette smoking was also associated with byssinosis, asthma, blood phlegm, and chronic bronchitis. Additionally, the illness-associated work absence was higher in workers of dusty worksites (sections) than those working in the less dusty sites. Work absences were significantly less common among workers who used face masks than among those who did not.

Local studies reported a high prevalence of byssinosis in Karachi (ranging from 19% to 35%) and Faisalabad (16.4%) [5, 6, 8, 18, 19, 35]. Generally, workers engaged in the dustier work sections, e.g., opening, blow room, and card room of textile mills, reported an elevated risk of byssinosis than those who work in less dusty sections, e.g., simplex, ring, and auto cone [6, 18, 19]. Similar results were reported in other studies. An Indian study found that byssinosis was as high as 30% and 38% in the blow room and card room, respectively [6].

The textile spinning process is divided into many stages (sections). The early sections, e.g., bale opening, blow room, and card room, contain high levels of endotoxins [10, 13, 14, 27, 30, 36]. Endotoxins are typically considered one of the significant reasons for byssinosis [16]. Several studies reported that most workers in the early sections of spinning processes showed byssinosis due to the high concentration of respirable cotton dust with endotoxin. Therefore, the persons who work in these sections can have a definite association with byssinosis and respiratory diseases [11, 14, 37, 38]. Conversely, subsequent work sections such as simplex, ring frame, and auto cone pose an average risk of byssinosis due to less dustiness [5, 6, 28, 36, 38]. In addition, many studies around the globe found a relationship between dust pollution and respiratory problems such as chronic cough, bronchitis, and asthma in textile workers [5–7, 9–14, 21, 27, 39].

Smoking is considered an important risk factor for byssinosis among textile mill workers. Similar to our results, previous studies showed that the risk of byssinosis was significantly higher in workers who smoke than in those who do not smoke [11, 13, 39]. Research has shown that cigarette smoking multiplies the effect of cotton dust exposure and elevates the risk of developing Byssinosis [38]. These results were supported by a study conducted in India showing higher symptoms of byssinosis among smokers [39]. These findings strengthen the results of our study that smoking serves as an additive to cotton dust exposure, aggravates the effects of dust pollution in the work environment, and causes an increased risk of respiratory diseases.

Work-related illness is typically associated with functional limitations. One functional limitation is the loss of workdays [6]. Occupational diseases and injuries are preventable by taking control measures at sources of pollution and by educating workers to adopt safety gadgets to avoid health hazards in the workplace [6, 40, 41]. Hence, workers and mill management can play a pivotal role in achieving compliance with occupational safety and health standards and reducing workplace health hazards [41]. Regarding workers, the results showed that despite the free provision of face masks, only a small percentage of workers were using face masks, whereas the use of earplugs was almost nonexistent in our sample. It must be noted that similar results have been found in other developing countries [8, 11–13, 36, 38–40, 42]. Research has shown that the control measures are not satisfactory in developing countries, and as a result, occupational diseases and injuries in textile mills are relatively higher.

The results of the current study state that the use of masks may have little effect on preventing respiratory illnesses. Rather, environmental and factory characteristics are real factors that determine the illness. However, the use of a mask may reduce the severity of the illness; in particular, the use of masks reduces sickness absences and visits to the doctor. It is emphasized here that the use of masks was higher among female workers than male workers. Furthermore, female workers are significantly less frequent visitors to doctors than male workers (although Mitigating Activities Function is not part of the analysis, the results stated that visits to a doctor are significantly more frequent for male workers than female workers, and this may be attributed to the use of masks). Whatever the underlying reasons for the use of face masks, the use of masks turns out to be beneficial in terms of reducing the functional limitations of the illness.

Regarding mill management, these results showed that the cost of work lost due to illness could be averted by mandating safety masks in the workplace. Apart from reducing work hours lost among mask users, the use of masks may also improve the safety culture in textile mills [6]. For example, a study in Karachi, Pakistan, showed that mill management strictly enforced the use of safety masks, resulting in fewer respiratory problems among workers [19]. Undoubtedly, preventive measures are the most cost-effective tool to reduce diseases and accident rates in the workplace [40, 41,43]. This is particularly important in the case of Pakistan, where the promulgation of cotton dust standards seems unlikely to happen in the period ahead. Therefore, the only available option in the near future is the use of protective measures by workers [6].

Meanwhile, it is crucial to understand the barriers and motivations for adopting face masks by workers, which may be due to the high temperature that dissuades workers from consistently using face masks [40, 41,43]. This may also be due to gender differences in safety perception. Generally, male workers are assumed to be less cautious about workplace safety than their female counterparts [42]. Alternatively, the higher use of face masks among female workers may be due to cultural aspects, as most Pakistani women cover their face and head with a shawl “locally called dupatta”. Therefore, the higher use of face cover by female workers may be due to cultural factors rather than safety concerns [6]. It could be a good area for researchers to explore the barriers to and motivations for workers to adopt face masks in the future.

### Limitations of the study

Although this study provides valuable insights into the association between cotton dust exposure and respiratory illness in the textile industry, it does have some limitations. This study recruited only current industry workers, which did not allow us to explore the perspective of ex-factory workers. In addition, the study relied on self-reported information for assessing cotton dust exposure and respiratory diseases, which is not an accurate measurement method. Furthermore, self-reported symptoms cannot be fully defined as clinical diagnoses. Hence, self-reported information can generate biased estimates about symptoms. Therefore, we propose that the results may be validated by using valid instruments for dust measurement and the clinical diagnosis of respiratory symptoms. However, the study estimates closely resemble clinical studies, and the reported severity of the symptoms of chest tightness on the opening day of the week, which becomes less severe on other days, are clear indications that reported symptoms are likely to be Byssinosis [6, 30, 34]. Furthermore, we used a simplified version of the Health Production Function, which makes no distinction between one episode of illness of two days or two separate illnesses of a single day each. Of course, this simplification ignores the severity of the illness. Furthermore, a richer specification of health production functions incorporating types of illness and severity of the symptoms can better explain the burden of illness.

## Conclusion

Cotton dust exposure is a major risk factor for many respiratory disorders, including byssinosis. The illness caused by cotton dust adversely affects workers’ health and quality of life. The results showed that textile workers face multiple respiratory symptoms, including chronic cough, phlegm, blood with phlegm, bronchitis, and byssinosis. Dusty work sections and smoking cigarettes were potential factors of respiratory illnesses. The workers use fewer safety gadgets irrespective of their level of education. However, the use of masks is higher among female workers than male workers. The textile workers employed in dusty sections take more days off. However, the number of work hours lost can be decreased if workers use face masks.

The findings of this study have some important policy implications. The disease varies across the work sections, showing an evident link between respiratory diseases and dust concentration at worksites. This information is crucial in establishing dust concentration standards within the textile industry. It is imperative for factory management to enforce the use of personal protective equipment. Alternatively, raising awareness among workers can serve as a motivating factor for mask use. It must be noted that implementing personal protective equipment is the most cost-effective strategy to reduce the occupational health burden in textile mills.

### Supplementary Information

**Additional file 1: Supplementary Table.** Definition of study variables concerning statistical analysis (*N* = 206).

## Data Availability

The data and survey questionnaire are publicly available from the South Asian Network for Development and Environmental Economics (SANDEE), which can be accessed at www.sandeeonline.org. The manuscript of the research is publicly accessible as a working paper on the following link: chrome-extension://efaidnbmnnnibpcajpcglclefindmkaj/http://www.sandeeonline.org/uploads/documents/publication/1110_PUB_WP123_17_Muhammad_Khan.pdf.

## Abbreviations

APTMA: All Pakistan Textile Mills Association
GDP: Gross domestic product
PKR: Pakistani Rupees
