# Supplementary material for: The health and economic burden of dust pollution in the textile industry of Faisalabad, Pakistan
Source: J Egypt Public Health Assoc. 2024 Jan 29;99:3. doi: 10.1186/s42506-024-00150-2 (PMC10822829; doi:10.1186/s42506-024-00150-2)
Supplement: Supplementary file 1 — Additional file 1: Supplementary Table. Definition of study variables concerning statistical analysis (N = 206). [file 42506_2024_150_MOESM1_ESM.docx]

**Supplementary Table.** Definition of study variables concerning statistical analysis (N = 206)

| **Variables** | **Definition** |
| --- | --- |
| *Personal Characteristics (G)* | |
| Gender | 1 if the worker is male |
| Age | In Years |
| Smoking index | Frequency of cigarette consumption per day multiplied by years of smoking |
| Marital status | 1 if the worker is married; 2 if the worker is single; 3 if the worker is divorced/separated; 4 if worker is the widow |
| Education | Total years of education |
| *Income Related (I)* | |
| Wage per month | In Pakistani Rupees (PKR) |
| Daily wage workers | 1 if the worker is working as a daily wage earner |
| Casual employee | 1 if the worker is working on a casual/contract basis |
| Permanent employee | 1 if the worker is permanently employed |
| *Averting Activities (A)* | |
| Use of mask | 1 if a textile worker is using a mask |
| *Dose‒Response Function (D)* | |
| Asthma | 1 if the worker is medically diagnosed (through a physical exam) with asthma. |
| Chronic cough | 1 if the worker often coughs in the morning and during the day ("often means more than 5 days per week"). |
| Bronchitis | 1 if the worker is diagnosed (through a physical exam) with Bronchitis by the medical practitioner. |
| Blood phlegm | 1 if the worker occasionally observes blood with mucus. |
| Byssinosis | 0 if no byssinosis; 1 if Grade ½ byssinosis; 2 if Grade 1 byssinosis; 3 if Grade 2 or 3 byssinosis |
| *Factory or Environmental Characteristics (C)* | |
| Ring section | 1 if the worker is employed at the ring section |
| Opening section | 1 if the worker is employed at the opening section |
| Blow room section | 1 if the worker is employed at the blow room section |
| Cardroom section | 1 if the worker is employed at the card room section |
| Samplex section | 1 if the worker is employed at the samplex section |
